# The influence of hashed fingerprints density on the machine learning methods performance

**DOI:** 10.1186/1758-2946-5-S1-P25

**Published:** 2013-03-22

**Authors:** Sabina Smusz, Rafał Kurczab, Andrzej J Bojarski

**Affiliations:** 1 Department of Medicinal Chemistry, Institute of Pharmacology Polish Academy of Sciences, Kraków, 31-343, Poland; 2 Faculty of Chemistry, Jagiellonian University, Kraków, 30-060, Poland

## 

Computational techniques have become a vital part of today's drug discovery campaigns. Among a wide range of tools applied in this process, machine learning methods can be distinguished. They are used for instance in virtual screening (VS), where its role is to identify potentially active compounds out of large libraries of structures [1].

In order to enable the application of various learning algorithms in VS tasks, an appropriate representation of molecules is needed. One of the solutions comes from the hashed fingerprints, encoding the information about the structure in a form of a bit string [2].

Both length and density (the percentage of 1's) can be modified during hashed fingerprint generation, which (as it was already proved) influence the similarity searching process [3]. The aim of our study was to examine the impact of such fingerprint density on the performance of machine learning methods. A series of bit strings with different density values and of various lengths was generated by means of the RDKit software [4]. They were tested in classification tests of 5-HT_1A _ligands, with the use of a set of algorithms (Naïve Bayes, SMO, Ibk, Decorate, Hyperpipes, J48 and Random Forest), in order to determine an optimal values of the variables for machine learning experiments.
